# Non postoperative biloma in Mauritania: case report and literature review

**DOI:** 10.11604/pamj.2018.31.237.17607

**Published:** 2018-12-20

**Authors:** Ahmedou Moulaye Idriss, Yahya Tfeil, Jiddou Sidi Baba, Ahmedou Mohamed Abdallahi, Ahmed Bezeid

**Affiliations:** 1 Department of Surgical Specialties, Faculty of Medicine, Nouakchott Al Aasriya University, Mauritania; 2 National Center Hospital, Nouakchott, Mauritania

**Keywords:** Biloma, imaging, CT-scan, fluid collection, drainage, cholelithiasis

## Abstract

Biloma is used to describe abnormal accumulation of bile outside biliary tract. It is a very rare condition with extrahepatic diffused or encapsulated collection of bile, mostly post-operative or post traumatic. A 72-year-old woman was referred to our hospital with acute abdominal pain located in right upper quadrant. Clinical examination suspected abdominal collection. Imagery (ultrasound and computed tomography scan) demonstrated a large well-defined intra-abdominal collection. Percutaneous ultrasound guided drainage of abdominal collection revealed a bile fluid. Drain was removed a week later and complete resolution of symptoms was obtained in two weeks. Even in the absence of specific diagnostic indications, radiological images may play a key role in the evaluation of suspected biloma in patients with appropriate medical history and clinical characteristics.

## Introduction

Biloma is a term used to describe the occurrence of biliary fluid outside the biliary tract. In 1889, there was a [1] reported study of the first case with patient kicked by horse. Eighty years later the word Biloma was introduced by Gould L. [2], who described subject with extra hepatic bile leakage in the upper right quadrant of the abdominal trauma from fighting. Most commonly, bilomas occur in the extra hepatic space with relatively few instances of hepatic subcapsular bilomas. On literature review, bilomas are commonly caused by iatrogenic injury or resulting from abdominal trauma [3, 4]. Iatrogenic damage to the biliary system is mostly associated to laparoscopic cholecystectomy [5]. Spontaneous rupture of the biliary tract is reported, and occasionally biloma is rarely observed or being associated [3, 5]. Locally chronic inflammation related to detergent bile acids activity causes adhesions, leading to a possible loculated appearance of the collection [4]. The diagnosis is suspected on the basis of the clinical history and usually weeks is required to make the correct diagnosis. In patients it is possible to note clinically; RUQ abdominal pain or distended abdomen, jaundice (choledocholithiasis), peritoneal irritation symptoms or complicated peritonitis with more severe sepsis, [3-6]. We describe a clinical case managed with percutaneous drainage and review of the literature.

## Patient and observation

**Figure 1:**
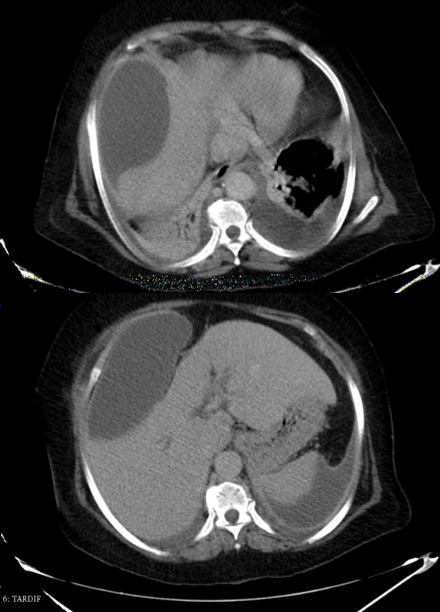
Abdominal CT demonstrating a large right hepatic subcapsular collection (black arrow)

**Figure 2:**
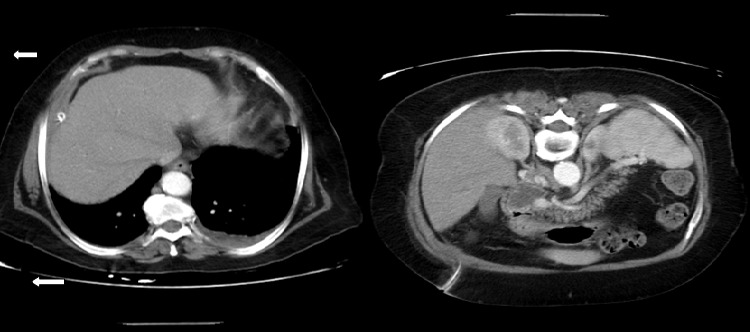
Abdominal CT showing drain (white arrow) with resolution of the biloma

A 72-year-old female previously healthy, was referred to our teaching hospital suffering from acute abdominal pain located in the right hypochondrium 6 days before. The patient was giving a history of minor abdominal trauma six months back for which monitoring and medical treatment were performed in a peripheral health center. However, the physical examination revealed the presence of pain and palpable mass in the epigastrium and upper right quadrant. Nausea and vomiting, respiratory gene and moderate jaundice were noted on arrival at the emergency room. Laboratory tests documented leukocytosis (12000/mL) with neutrophilia. Hemoglobin, serum electrolytes, indices of cholestasis, liver enzymes, kidney function, lipasemia, amylasemia and blood glucose were normal. Abdominal ultrasound and computed tomography scan findings included a large well-defined intra-abdominal (right quadrant) collection (Figure 1) overlying the suprahepatic and right subphrenic regions (max axial 15 cm), gallstones with extra hepatic bile duct moderate dilation without being able to identify common bile duct stones, and the inter-hepato-diaphragmatic fluid collection exerting a mass effect on the liver. In addition bilateral pneumonic pleuro pneumopathy and peritoneal effusion were observed. After percutaneous drainage and antibiotic therapy, control CT scan showed the disappeared lesion (Figure 2) and the patient obtained complete resolution of symptoms. The drainage was removed a week later. One month later, CT-scan revealed cholelithiasis and the patient underwent cholecystectomy with removal of common bile duct stone.

## Discussion

It is named biloma any bile collection encapsulated or not in the abdomen, as a consequence of the biliary leak, from biliary tract, mainly of the extrahepatic segment or directly from the canaliculi. The most common etiology is surgical, endoscopic or traumatic lesions [7]. The literature reports a very low prevalence of fluid biliary collection, and the majority of biloma studies have been based on case reports. Most biloma develops following surgery and abdominal trauma [3, 5, 8]. As the preference for laparoscopic biliary surgery is increasing, the occurrence of biloma due to iatrogenic perforation increased from 0.1% to 1.5% [4]. During the last 10 years, Sung-Bum *et al.* [9] reported 11 cases of non-traumatic perforation of the bile ducts. In addition to post-operative and post traumatic injuries, there is also duodenal diverticulum [7] the lost stones in abdominal cavity after a cholecystectomy [10] pancreatic cancer [6] choledochal diverticulum perforation [11] and chronic pancreatitis [9] as etiological factors. It is difficult to determine the etiology of the event, since sometimes it is not possible to identify the site of the lesion. Spontaneous perforation of the bile duct in adults' unrelated to iatrogenic injury or severe trauma is extremely rare and is more often seen in infants and children [12-14].

Although the pathophysiology of spontaneous biloma remains to be clarified [15], one study suggested contributing factor is an increased intraductal pressure due to obstructive lesions or infarctions of any part of the biliary tree [16]. The most frequent cause of spontaneous biloma is choledocholithiasis [15, 16]. However, several causes of spon¬taneous perforation of bile duct have been seen due to ero¬sion caused by biliary stones that injured the duct wall; increased intraductal pressure due to an obstruction of the distal bile duct (by stones, carcinomas, or a reflux spasm of the sphinc¬ter of Oddi); thrombosis of a vessel supplying the bile duct wall; intramural infection of the duct as a result of cholangitis; regurgitation of pancreatic secretions into the bile duct; diverticulitis of the bile duct; and acute pancreatitis [17-19]. Bilomas are generally localized in the right upper quadrant of the abdomen, neighboring the right hepatic lobe [16]. Our patient presented with localized fluid in the right hypochondrium and suggested a localized perihepatic collection causing extrinsic liver compression. The presence of jaundice and cholelithiasis in present case, suggested the diagnosis of biloma. Guided percutaneous puncture, as performed in this case with US, is useful for both the diagnosis and the treatment of bilomas [3, 6, 7, 16]. In our patient, 7weeks later removal of the stone was performed by open surgery. The conservative treatment of bilomas includes intravenous hydration and antibiotic therapy (particularly in cases of sepsis) is recommended by some authors [3, 4, 5].

## Conclusion

Biliary tract lesions remain a challenge, both diagnostic and therapeutic. Fortunately, with the advent of new technologies, this has been facilitated, so that today patients present adequate recovery and without sequelae, in most cases. Percutaneous treatment should be considered as the first-line option for patients with symptomatic biloma.

## Competing interests

The authors declare no competing interests.
